# Bronchiectasis in Systemic Autoimmune Rheumatic Diseases: Clinical Significance and Diagnostic Challenges

**DOI:** 10.3390/diagnostics16213550

**Published:** 2026-10-28

**Authors:** Maria Chernovsky, Renata Greco, Micol Pividori, Giorgio Monteleone, Michael Hughes, Paola Confalonieri, Francesco Salton, Eugenio Capparelli, Yukai Wang, Giulia Bandini, Marco Confalonieri, Maria Chianese, Umberto Semenzato, Barbara Ruaro

**Affiliations:** 1 Pulmonology Unit, Department of Medical Surgical and Health Sciences, Hospital of Cattinara, Univesity of Trieste, 34149 Trieste, Italy; 2 Center for Interstitial and Rare Lung Diseases, Department of Pneumology, Ruhrlandklinik, University Hospital Essen, University of Duisburg-Essen, 45147 Essen, Germany; 3 Centre for Musculoskeletal Research, Division of Musculoskeletal and Dermatological Science, School of Biological Sciences, Faculty of Biological Medicine and Health, The University of Manchester, Manchester Academic Health Science Centre, Stopford Building, Oxford Road, Manchester M13 9PL, UK; 4 Department of Rheumatology, Northern Care Alliance NHS Foundation Trust, Salford Care Organisation, Salford M6 8HD, UK; 5 NIHR Manchester Biomedical Research Centre, Manchester University NHS Foundation Trust, Manchester M8 5RB, UK; 6 Rheumatology Unit, ASST Ovest Milanese, Legnano Hospital, Legnano, 20025 Milan, Italy; 7 Department of Rheumatology and Immunology, Shantou Central Hospital, Shantou 515041, China; 8 Department of Experimental and Clinical Medicine, Division of Internal Medicine, Azienda Ospedaliero Universitaria Careggi, University of Florence, 50134 Florence, Italy; 9 IRCCS AOU Ss Antonio e Biagio e Cesare Arrigo di Alessandria, 15121 Alessandria, Italy; 10 Respiratory Medicine, Department of Cardiac, Thoracic, Vascular Sciences and Public Health, University of Padova, 35128 Padova, Italy

**Keywords:** bronchiectasis, rheumatic diseases, rheumatoid arthritis, traction bronchiectasis, connective tissue disease-associated interstitial lung disease, respiratory infections, immunosuppression, disease activity

## Abstract

Bronchiectasis has been reported in several systemic autoimmune rheumatic diseases, but published estimates are difficult to interpret because many studies count any bronchial dilatation on computed tomography (CT). This is particularly problematic in connective tissue disease-associated interstitial lung disease (ILD), where traction can enlarge the airways without producing the clinical syndrome of bronchiectasis. We reviewed evidence in rheumatoid arthritis, Sjögren disease, anti-neutrophil cytoplasmic antibody-associated vasculitis, systemic sclerosis, systemic lupus erythematosus, and inflammatory myopathies, with attention to imaging definitions, respiratory symptoms, infection, outcomes, and treatment. The association with a distinct airway disorder is best established in rheumatoid arthritis, Sjögren disease, and myeloperoxidase-ANCA-positive vasculitis. In systemic sclerosis and systemic lupus erythematosus, large administrative cohorts suggest an increased occurrence of bronchiectasis, but most data do not allow for confident separation from traction bronchiectasis or other causes. In inflammatory myopathies, reported bronchial dilatation is mainly described within fibrotic ILD. In practice, interpretation should combine high-resolution CT morphology and distribution with cough and sputum, exacerbation history, microbiology, and the presence and extent of ILD; traction and non-traction changes may coexist. Bronchiectasis-specific care should follow established guidelines, while immunomodulatory therapy is selected for the rheumatic disease with explicit consideration of infection risk. No trial has identified a preferred antirheumatic regimen for patients with bronchiectasis, and it remains unknown whether better control of systemic disease changes bronchiectasis outcomes. Studies using consistent clinical and radiological definitions are needed. In mixed connective tissue disease, available evidence is insufficient to characterise bronchiectasis independently of ILD. Seven disease categories are reviewed in total.

## 1. Introduction

Bronchiectasis is a heterogeneous chronic respiratory disease defined by permanent bronchial dilatation on CT together with a compatible clinical syndrome, usually including cough, sputum production, and recurrent respiratory infections [1]. It may result from infectious, immune-mediated, genetic, or structural processes. In the European Bronchiectasis Registry (EMBARC), rheumatoid arthritis and other connective tissue diseases accounted for approximately 4.8% of assigned aetiologies among people already diagnosed with bronchiectasis [2].

Among systemic autoimmune rheumatic diseases (SARDs), the lungs are frequently involved, with abnormalities of the airways, parenchyma, pleura, and pulmonary vasculature [3,4]. The term “bronchiectasis” is used both for a radiological sign and for a clinical disease. Fibrotic retraction in connective tissue disease-associated interstitial lung disease (ILD) can dilate bronchi and bronchioles, producing traction bronchiectasis that reflects architectural distortion. Conversely, bronchiectasis may be a separate airway manifestation. The distinction is not merely semantic: minor bronchial dilatation may be clinically silent, whereas patients with relatively limited radiological change may still have troublesome sputum and frequent exacerbations.

Several mechanisms may connect rheumatic disease with bronchiectasis, and their relative importance probably differs between diseases. Chronic mucosal immune activation, autoantibody-associated airway injury, impaired mucociliary clearance, recurrent infection, sicca-related secretion abnormalities, aspiration, and immunosuppressive treatment have all been proposed; these mechanisms are discussed in the Section Shared and Disease-Specific Mechanisms and the disease-specific sections below. Bronchiectasis itself is heterogeneous, with different clinical phenotypes and biological mechanisms [5]. Sequencing studies have shown that complex airway microbial communities may persist with little change after antibiotic treatment of exacerbations [6]. In established bronchiectasis, persistent infection, impaired clearance, and neutrophilic inflammation can reinforce one another and sustain airway damage [5,7,8]. This process can occur independently of fibrosis, although non-traction bronchiectasis and traction bronchiectasis may coexist.

These distinctions influence both diagnosis and management. Clinically relevant bronchiectasis may require sputum surveillance, airway-clearance treatment, and prevention of recurrent exacerbations, while rheumatologists must also consider infection risk when choosing glucocorticoids, disease-modifying antirheumatic drugs (DMARDs), biological agents, or targeted synthetic therapies [7,9,10,11,12,13]. This review therefore examines disease-specific evidence with particular attention to how bronchiectasis was defined, whether ILD-related traction was excluded, and what is known about respiratory infections, outcomes, and treatment.

Compared with previous reviews, this article specifically examines how the definition of bronchiectasis—radiological, clinical, or administrative—determines the prevalence estimate for each disease, and provides a systematic appraisal of whether ILD-related traction was excluded in each study [3,4,14,15,16]. This distinction has direct diagnostic and therapeutic implications that are discussed throughout. Seven disease groups are covered, including mixed connective tissue disease.

## 2. Materials and Methods

This structured narrative review was based on searches of PubMed/MEDLINE, the Web of Science Core Collection, and the Cochrane Library, focusing primarily on English-language publications from January 2021 through July 2026. Search terms combined bronchiectasis or airway disease with rheumatoid arthritis, systemic sclerosis, systemic lupus erythematosus, Sjögren disease, ANCA-associated vasculitis, inflammatory myopathies, or mixed connective tissue disease. For publications before 2021, inclusion was selective and limited to seminal studies, landmark cohorts, and earlier work providing the principal or only available evidence for a specific disease or clinical question. Reference lists of relevant reviews and selected articles were screened specifically to identify these earlier sources. We considered systematic reviews, meta-analyses, and original observational or registry studies reporting prevalence, imaging, microbiology, exacerbations, outcomes, or treatment-related events. Evidence was synthesised narratively, with particular attention to how bronchiectasis was defined and whether bronchial dilatation reflected traction within interstitial lung disease or a distinct airway manifestation (Supplementary Material).

Studies prior to 2021 were retained if they represented the principal or sole source of evidence for a specific disease-bronchiectasis association (e.g., Soto-Cardenas et al. [17] for Sjögren disease; Néel et al. [18] for ANCA-associated vasculitis), or if they defined natural history in landmark cohorts not replicated by later work, or if they provided imaging or microbiological data unavailable elsewhere. The use of a large language model (Claude, Anthropic, San Francisco, CA, USA) for drafting and language editing is declared in accordance with the journal’s AI-use policy; all content was critically reviewed and approved by all authors, who take full responsibility for scientific accuracy and integrity.

### Shared and Disease-Specific Mechanisms

Several mechanisms may contribute to the development or perpetuation of bronchiectasis in SARDs, and their relative importance differs between diseases. Chronic mucosal immune activation is particularly relevant in rheumatoid arthritis and Sjögren disease, where lymphocytic infiltration of the bronchial mucosa, impaired mucociliary clearance, and sicca-related secretion abnormalities can predispose patients to recurrent bacterial colonisation and airway injury. In RA, autoantibody-associated airway injury has been proposed: anti-citrullinated protein antibodies (ACPAs) have been detected in bronchial secretions and may be produced locally in response to mucosal citrullination, contributing to immune activation before the onset of joint disease [14,15]. In AAV, MPO-ANCA positivity and neutrophil extracellular trap (NET) formation may sustain airway neutrophilia and structural damage independently of systemic vasculitis [19].

**Figure 1:**
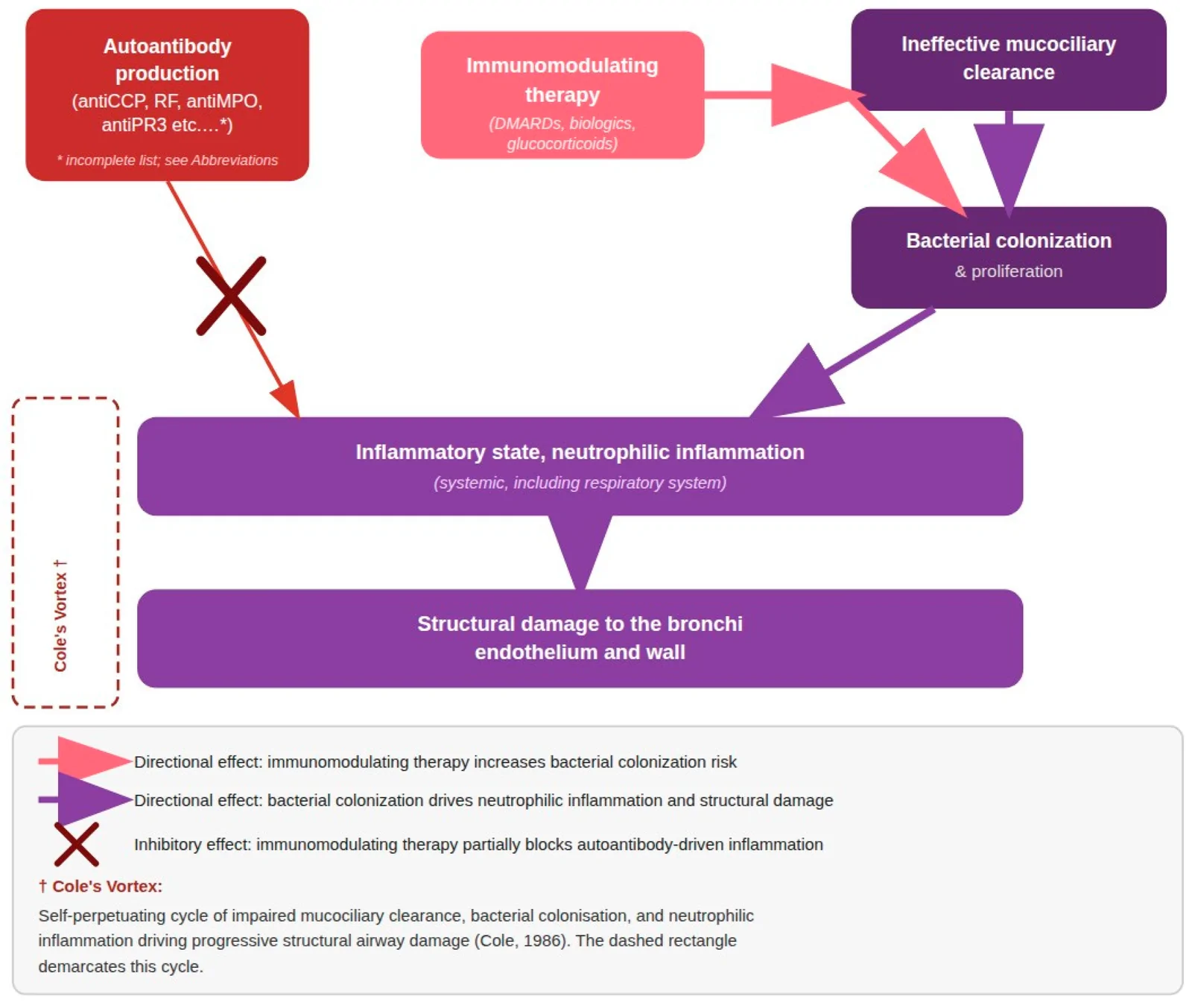
A flowchart exemplifying the proposed bidirectional interactions between bronchiectasis and rheumatologic diseases and the latter’s role in propagating the classic “Cole’s Vortex” model in bronchiectasis. Autoantibody production favours an inflammatory state in a systemic manner, including the respiratory system, where neutrophilic inflammation is a key factor in creating structural damage to the bronchi lining and wall. Immunomodulating drugs often used in rheumatologic diseases decrease the level of systemic inflammation, but increase the risk of bacterial proliferation and colonization, which in and of itself favours an increase in neutrophilic inflammation. Cole’s Vortex refers to the self-perpetuating cycle of impaired mucociliary clearance, bacterial colonisation, and neutrophilic inflammation originally described by Cole (1986) as a model of bronchiectasis perpetuation; the dashed rectangle in the figure demarcates this cycle. Single-headed arrows indicate directional (one-way) effects; the cross symbol (✕) indicates an inhibitory or blocking effect. The ellipsis (…) indicates that the list of autoantibodies shown is non-exhaustive: other disease-specific autoantibodies exist beyond those depicted. Abbreviations: RF, Rheumatoid Factor; antiCCP, anti-citrullinated protein antibodies; antiMPO, anti-Myeloperoxidase; antiPR3, anti-Proteinase 3; DMARDs, disease-modifying antirheumatic drugs.

Immunosuppressive treatment represents a shared mechanism with dual effects: by reducing systemic and airway inflammation it may modulate disease activity in bronchiectasis, while simultaneously increasing susceptibility to bacterial colonisation and pulmonary infection. This bidirectional interaction is illustrated schematically in Figure 1, which depicts how autoantibody production, systemic inflammation, immunomodulatory treatment, bacterial proliferation, neutrophilic inflammation, and structural airway damage form a connected cycle—the proposed rheumatic contribution to Cole’s Vortex model of bronchiectasis perpetuation. The mechanisms depicted are pathophysiological hypotheses supported by observational data; causal relationships remain to be established in prospective studies (Figure 1).

Aspiration and gastro-oesophageal reflux are proposed mechanisms in SSc (oesophageal dysmotility) and Sjögren disease (hiatus hernia), but their independent contribution to bronchiectasis cannot be separated from other factors in the available data [17,20]. Recurrent respiratory infection, which is both a consequence and a driver of bronchiectasis, is discussed in each disease-specific section below.

## 3. Bronchiectasis and Traction Bronchiectasis

A CT report of bronchiectasis is not sufficient, on its own, to diagnose bronchiectasis as a clinical disease. HRCT should be assessed for bronchial enlargement, lack of tapering, and peripheral airway visibility, but also for distribution, wall thickening, mucus plugging, tree-in-bud opacities, and air trapping [1]. These findings need to be considered alongside chronic cough, sputum production, recurrent infection, and exacerbations. Mild radiological dilatation may have little clinical relevance, while symptoms and disease burden can be relevant even when bronchial dilatation is limited.

**Figure 2:**
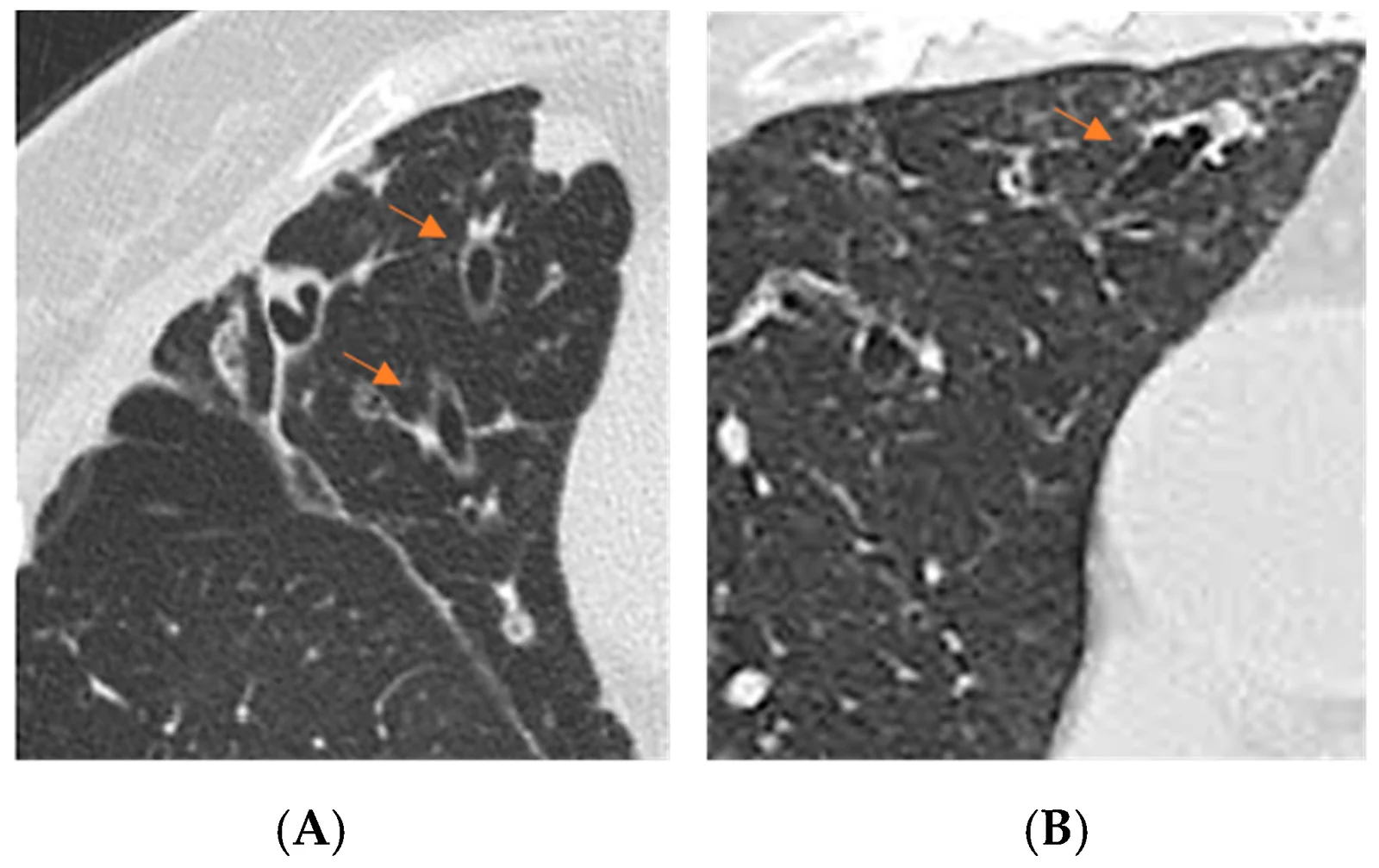
Panel (**A**): This image illustrates bronchiectasis associated with Sjögren’s syndrome. Panel (**B**): This image depicts traction bronchiectasis-related connective tissue disease-associated ILD. Orange arrows highlight the areas of bronchiectasis.

Traction bronchiectasis is suggested when dilated and distorted bronchi lie within reticulation, volume loss, honeycombing, or other fibrotic architectural distortion. Bronchiectasis as a separate airway manifestation is more likely when dilatation extends beyond fibrotic regions and is accompanied by airway-centred abnormalities or a compatible clinical syndrome. Representative examples of bronchiectasis associated with Sjögren’s syndrome and connective tissue disease-associated ILD are shown in Figure 2.

The 2022 international consensus criteria for the radiological and clinical diagnosis of bronchiectasis in adults [1] define the radiological threshold as a broncho-arterial (BA) ratio 1.0 or greater on HRCT, measured using either the inner or outer airway diameter, with the caveats that age over 65 years and technical factors may affect this ratio [21]. Additional radiological features include lack of bronchial tapering and visibility of airways in the periphery. These radiological findings must be accompanied by a compatible clinical syndrome—defined as at least two of the following: cough on most days of the week, sputum production on most days of the week, and a history of exacerbations—to establish a diagnosis of bronchiectasis as a disease rather than an incidental imaging finding.

**Figure 3:**
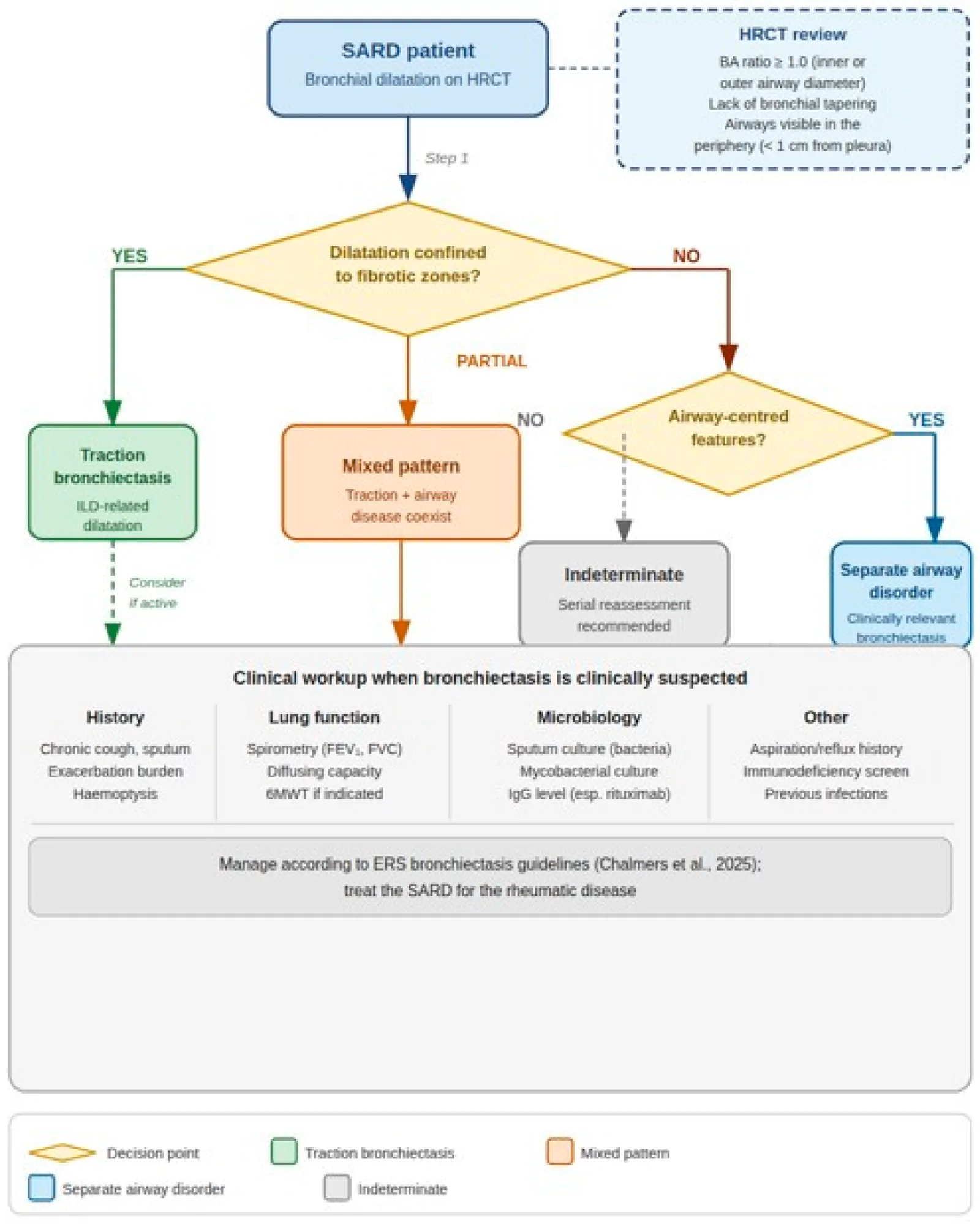
Example of diagnostic algorithm for bronchial dilatation in systemic autoimmune rheumatic diseases [7]. Abbreviations: 6MWT, six-minute walk test; BA, broncho-arterial; FEV_1_, forced expiratory volume in 1 s; FVC, forced vital capacity; HRCT, high-resolution computed tomography; IgG, immunoglobulin G; ILD, interstitial lung disease; SARD, systemic autoimmune rheumatic disease.

Applying these criteria in SARD patients requires additional caution. Fibrotic ILD distorts the lung architecture and compresses pulmonary vasculature, which may artifactually increase the BA ratio in traction bronchiectasis. Direct review of HRCT by an experienced radiologist is therefore preferable to reliance on the BA ratio alone when fibrosis is present. A practical approach is outlined in Figure 3: if bronchial dilatation is confined to areas of fibrotic reticulation, honeycombing, or volume loss, traction bronchiectasis should be considered; if dilatation extends into non-fibrotic lung with accompanying airway-centred features (wall thickening, mucus plugging, tree-in-bud nodularity), a separate airway disorder should be suspected; and when both patterns coexist, both should be documented and managed accordingly.

Traction bronchiectasis, although discussed here primarily as a source of diagnostic misclassification, is not clinically neutral. In connective tissue disease-associated ILD, the extent and severity of traction bronchiectasis on HRCT have been independently associated with all-cause mortality, contributing to ILD staging beyond simple pattern classification [22]. Identifying traction bronchiectasis therefore matters both because it should not be confused with independent airway disease and because its severity reflects the degree of fibrotic lung remodelling with prognostic implications in its own right. The distinction drawn in this review is between two disease processes that each require appropriate assessment, not between relevant and irrelevant dilatation.

When clinically relevant bronchiectasis is suspected, assessment should include respiratory history and exacerbation burden, spirometry, and microbiological evaluation, including routine bacterial and mycobacterial sputum cultures when indicated [7]. Other contributors, including previous infection, immunodeficiency, aspiration, and reflux, should be investigated according to the clinical context [7]; reflux is common in bronchiectasis, but its causal role remains uncertain [23]. This evaluation determines whether bronchiectasis-specific management is needed in addition to ILD or rheumatological care.

### 3.1. Rheumatoid Arthritis

Rheumatoid arthritis (RA) has the best-supported association with bronchiectasis, although estimates vary with the method of ascertainment. Martin et al. reported a pooled prevalence of 18.7% (95% CI 13.7–24.3%) and a prevalence of 22.6% (95% CI 16.8–29.0%) in studies using HRCT [24]. These findings largely reflect radiological bronchiectasis rather than symptomatic disease. A separate meta-analysis illustrates different results: the pooled estimate was 2.69% (95% CI 1.63–4.42%) in studies using a clinical definition and 24.9% (95% CI 19.21–31.67%) in radiological studies [25]. In an older HRCT series of 50 patients, cylindrical bronchiectasis was present in 30% and air trapping in 32%, but these were imaging findings rather than estimates of symptomatic disease [26]. A more recent analysis of studies in which consecutive patients underwent HRCT estimated a prevalence of 8.2% (95% CI 1.6–14.7%) at the time of RA diagnosis; across studies, prevalence was 1.1 percentage points higher for each additional year of mean disease duration [27]. In a Korean nationwide cohort, RA was associated with a 2.12-fold higher risk of a new bronchiectasis code than matched controls, with a stronger association in seropositive RA [28]. In a cohort of 204 patients with early RA undergoing repeated structured respiratory assessment, the cumulative incidence of non-tractional airway dilatation was 8.8% at 4 years and 12.9% at 8 years [29]. However, the imaging endpoint included both bronchial and bronchiolar dilatation and also captured asymptomatic abnormalities, so these figures do not represent the incidence of clinically significant bronchiectasis. Comparisons between studies remain difficult because definitions differ and ILD-related traction bronchiectasis is not always distinguished from non-traction bronchiectasis.

The temporal relation between RA and bronchiectasis is variable. Bronchiectasis may precede joint disease, which has led to the hypothesis that chronic airway inflammation and mucosal citrullination can contribute to loss of immune tolerance [14,15]; in other patients, systemic inflammation, infection, or treatment may contribute later in the course of RA. In a case–control study that excluded ILD, 57 patients with isolated RA-associated bronchiectasis were compared with 360 patients with RA and no lung disease. Older age at RA onset, lower body mass index, and seropositivity were independently associated with bronchiectasis; the odds ratio for seropositive RA was 3.96 (95% CI 1.84–8.53) [30]. This finding supports an association between bronchiectasis and RA-related autoimmunity but does not establish a causal role for autoantibodies in the development of bronchiectasis.

The clinical consequences also depend on the comparator and on how lung disease is defined. Among people with established bronchiectasis in a Korean national cohort, coexisting RA was associated with higher mortality than bronchiectasis alone (adjusted HR 2.09, 95% CI 1.88–2.33) [31]. In contrast, an imaging-verified US cohort that excluded ILD found no statistically significant increase in all-cause mortality for isolated RA-associated bronchiectasis compared with RA without lung disease, although respiratory mortality was higher (subdistribution HR 3.41, 95% CI 1.05–11.0) [32]. A related analysis did not show a significant increase in overall serious infection for the bronchiectasis group, but pulmonary infections and organisms such as Pseudomonas and nontuberculous mycobacteria were more frequent [9]. A small single-centre cohort also identified bronchiectasis as a risk factor for severe pneumonia during biological DMARD treatment [10].

Bronchiectasis treatment should follow established respiratory guidelines and may include airway-clearance techniques, vaccination, sputum-guided treatment of exacerbations, and preventive antimicrobial strategies in selected patients [7]. Antirheumatic drugs treat RA, not bronchiectasis, and no randomised trial has identified a preferred DMARD for this overlap. In an older retrospective cohort of 47 patients with rheumatic disease and bronchiectasis, biological treatment and sputum colonisation were independently associated with lower respiratory tract infections (OR 8.7, 95% CI 1.7–43.4 and OR 7.4, 95% CI 2.0–26.8, respectively) [11]. The small sample and wide confidence intervals limit the precision of these estimates. In a two-centre observational study, most rituximab-treated patients had stable or fewer infective exacerbations, with fewer discontinuations for respiratory events than among patients receiving tumour necrosis factor inhibitors [12]. A much smaller retrospective study found no excess respiratory hospitalisation or death with JAK inhibitors compared with rituximab, but included both ILD and bronchiectasis [13]. Treatment decisions should therefore take account of previous infections, sputum microbiology, bronchiectasis severity, immunoglobulin levels, glucocorticoid exposure, and the need to control RA. Importantly, no study has directly tested whether better control of RA reduces bronchiectasis exacerbations or prevents bronchiectasis progression.

### 3.2. Systemic Sclerosis

Systemic sclerosis (SSc) commonly causes fibrotic non-specific interstitial pneumonia and, less often, usual interstitial pneumonia; both patterns may contain traction bronchiectasis [33]. Non-traction bronchiectasis can, however, occur independently of fibrosis. In the Australian Scleroderma Cohort Study, HRCT images from 256 participants were reviewed with traction bronchiectasis recorded separately. Bronchiectasis was present in 42 patients (16.4%) and was negatively associated with ILD (OR 0.32, 95% CI 0.14–0.76) [20]. This was an HRCT-selected cohort rather than a population sample, but it provides evidence that not all bronchial dilatation in SSc is a consequence of fibrotic traction.

A Korean nationwide study adds complementary longitudinal evidence. Among 4845 adults with SSc and 24,225 age- and sex-matched controls, a bronchiectasis code appeared in 5.3% and 1.9%, respectively, over a median of 6 years (adjusted HR 2.63, 95% CI 2.22–3.12) [34]. The database contained no HRCT images, respiratory symptoms, lung function, or microbiology, so the anatomical and clinical nature of these diagnoses could not be determined. A sensitivity analysis excluding people with an ILD code produced similar results, suggesting that recognised ILD did not account for the whole association, although subclinical or uncoded ILD remained possible.

Oesophageal dysmotility, reflux, and aspiration have been proposed as additional mechanisms in SSc. In the Australian cohort, however, oesophageal dysmotility was present in 95.7% of participants; because almost all patients were affected, the study could not determine whether dysmotility was associated with bronchiectasis [20]. Thus, aspiration remains clinically plausible but unproven as a cause. When bronchial dilatation is reported, its distribution in relation to fibrosis should be reviewed and correlated with sputum, recurrent infections, and microbiology before a separate diagnosis of bronchiectasis is made. No prospective study has shown that treating reflux or oesophageal dysfunction prevents bronchiectasis or changes its course in SSc.

### 3.3. Systemic Lupus Erythematosus

Evidence specific to bronchiectasis in systemic lupus erythematosus (SLE) has been sparse, with the pulmonary literature dominated by pleural disease, infection, haemorrhage, and ILD rather than a well-characterised chronic airway disorder [16]. A Korean nationwide cohort published in 2026 provides a large longitudinal estimate. It included 3368 adults with SLE and 13,472 matched controls followed for a median of 12.8 years. Bronchiectasis incidence was 374.8 versus 236.2 per 100,000 person-years, corresponding to an adjusted HR of 1.46 (95% CI 1.22–1.76); the result was similar after excluding people with another connective tissue disease [35].

Within the SLE cohort, a bronchiectasis diagnosis was also associated with higher all-cause and respiratory mortality and greater healthcare use [35]. These findings are clinically important, but they arise from diagnostic codes without central HRCT review, symptoms, anatomical distribution, microbiology, or detailed treatment data. They therefore establish an association with coded bronchiectasis, not a distinct SLE-related airway phenotype or a causal effect on mortality. Recurrent infection, previous inflammatory lung injury, immune dysregulation, and immunosuppressive treatment are all possible contributors. At present, respiratory assessment should be guided by symptoms and imaging, and no SLE treatment can be recommended specifically because bronchiectasis is present.

### 3.4. ANCA-Associated Vasculitis

Bronchiectasis is most consistently reported in myeloperoxidase (MPO)-ANCA-positive disease and microscopic polyangiitis (MPA). A 2024 systematic review and meta-analysis of 24 studies estimated a pooled prevalence of 19% (95% CI 13–27%) across ANCA-associated vasculitis (AAV), with estimates of 25% in MPA and 28% in MPO-ANCA-positive disease, compared with 13% in proteinase 3-ANCA-positive disease [19]. Female sex, peripheral neuropathy, MPO-ANCA positivity, and MPA were associated with bronchiectasis. Most studies were retrospective and imaging-based, and fibrotic ILD was not consistently separated, so the pooled estimate probably includes some traction bronchiectasis.

Several observations nevertheless support a distinct airway manifestation. Cylindrical bronchiectasis was the most common morphology, and bronchiectasis preceded AAV in 13.6–43.8% of reported cases, often by many years [19]. In a French cohort of 58 patients with MPA or granulomatosis with polyangiitis, bronchiectasis was found in 22 (37.9%); all 22 were MPO-ANCA positive, with more peripheral nerve and less renal involvement than in patients without bronchiectasis [18]. Chronic airway infection and neutrophil activation could plausibly contribute to autoimmunity, while vasculitis and its treatment may later aggravate airway injury, but these temporal associations do not prove causation.

In serial imaging studies, bronchiectasis generally remained stable or progressed despite immunosuppressive treatment [19,36,37]. Control of vasculitis should therefore not be assumed to reverse established airway damage. Several cohorts included in the meta-analysis also reported more respiratory infections among patients with bronchiectasis [19], which is particularly relevant during intensive immunosuppression. Management should combine appropriate induction or maintenance therapy for AAV with sputum cultures, airway clearance, vaccination, and pathogen-directed treatment when bronchiectasis is clinically active [7]. There is no evidence that a particular AAV regimen should be chosen solely because bronchiectasis is present.

The available evidence is concentrated in MPO-ANCA-positive disease and MPA. Granulomatosis with polyangiitis (GPA) and eosinophilic granulomatosis with polyangiitis (EGPA) are associated with distinct patterns of airway and lung involvement—pulmonary nodules in GPA, reflecting necrotising granulomatous inflammation, and eosinophilic airway inflammation with bronchial hyperresponsiveness in EGPA [36]—which differ from the cylindrical bronchiectasis pattern predominating in MPA. In a meta-analysis, bronchiectasis was less prevalent in GPA than in the other AAV subtypes [19], and reported prevalences across AAV vary widely between studies [37]; the relative contributions of fibrotic traction, prior infectious damage, and primary airway vasculitis are difficult to separate in retrospective series [19]. Prognosis data in AAV-associated bronchiectasis are conflicting: in a nationwide multicentre cohort, bronchiectasis preceding AAV was associated with a five-fold increase in the risk of death [36], whereas in a small MPA cohort survival did not differ significantly between patients with and without bronchiectasis, although in the chronic phase mortality appeared higher than with other pulmonary manifestations [38]. Bronchiectasis progression on serial imaging despite immunosuppression underscores that airway damage in AAV may be only partially modifiable by vasculitis treatment [19,36,37].

### 3.5. Sjögren Disease

Sjögren disease can affect both large and small airways independently of ILD. In an older cohort of 507 patients, CT was performed when clinically indicated in 120 patients and identified bronchiectasis attributable to Sjögren disease in 41 patients (8% of the whole cohort and 34% of those scanned) [17]. CT was not performed systematically, so conclusions regarding prevalence could not be made. All cases were cylindrical and 71% involved the lower lobes, supporting an airway pattern rather than a disease-specific radiological signature. Two small contemporary cohorts illustrate the variability of such estimates: bronchiectasis was reported in 3.3% in one clinical series, whereas bronchial wall thickening and bronchiectasis were recorded in 8 of 22 patients in an HRCT pilot study [39,40]. Their size and selection preclude population prevalence estimates.

More recent evidence comes from a multicentre study of 31 patients with Sjögren-associated airway disease and 62 matched controls without pulmonary involvement. Central CT review showed multilobar abnormalities in 72% and combined features of bronchiectasis and bronchiolitis in 60%; airflow obstruction was present in 32% at diagnosis [41]. Lung function remained broadly stable over a mean of 8.7 years, although airway abnormalities extended on serial CT in 41% over approximately 6 years. No patient developed Sjögren-associated ILD [41]. These findings suggest that bronchiectasis is not simply related to traction from fibrosis.

Mucosal dryness, impaired clearance, lymphocytic bronchiolitis, and recurrent infection may all contribute to bronchiectasis. In the older cohort, respiratory infections (56% vs. 3%) and pneumonia (29% vs. 3%) were more frequent in patients with bronchiectasis, and hiatus hernia was also more common (41% vs. 16%) [17]. The latter observation makes reflux or aspiration plausible in some patients but does not establish causality. No trial has identified an immunomodulatory treatment for Sjögren-associated bronchiectasis itself. Clinically relevant disease should be managed according to general bronchiectasis principles, alongside assessment of inflammatory airway or parenchymal involvement related to Sjögren disease [7].

### 3.6. Inflammatory Myopathies and Mixed Connective Tissue Disease

In polymyositis and dermatomyositis, the available literature describes bronchial dilatation mainly within fibrotic NSIP, UIP, or mixed NSIP/organising-pneumonia patterns [42,43]. In this setting it is best interpreted as traction bronchiectasis unless a separate non-fibrotic distribution and a compatible airway syndrome are shown. The very small series that report bronchiectasis in rare autoantibody-defined subgroups cannot establish the prevalence of an independent airway disease.

For mixed connective tissue disease, no contemporary disease-specific cohort identified in the search adequately characterised bronchiectasis as a clinical airway disorder. The literature is dominated by ILD, and neither the frequency nor the consequences of non-traction bronchiectasis can presently be estimated. Table 1 summarises the available disease-specific evidence and how clinically relevant bronchiectasis was separated from bronchial dilatation caused by fibrotic traction.

## 4. Discussion

**Figure 4:**
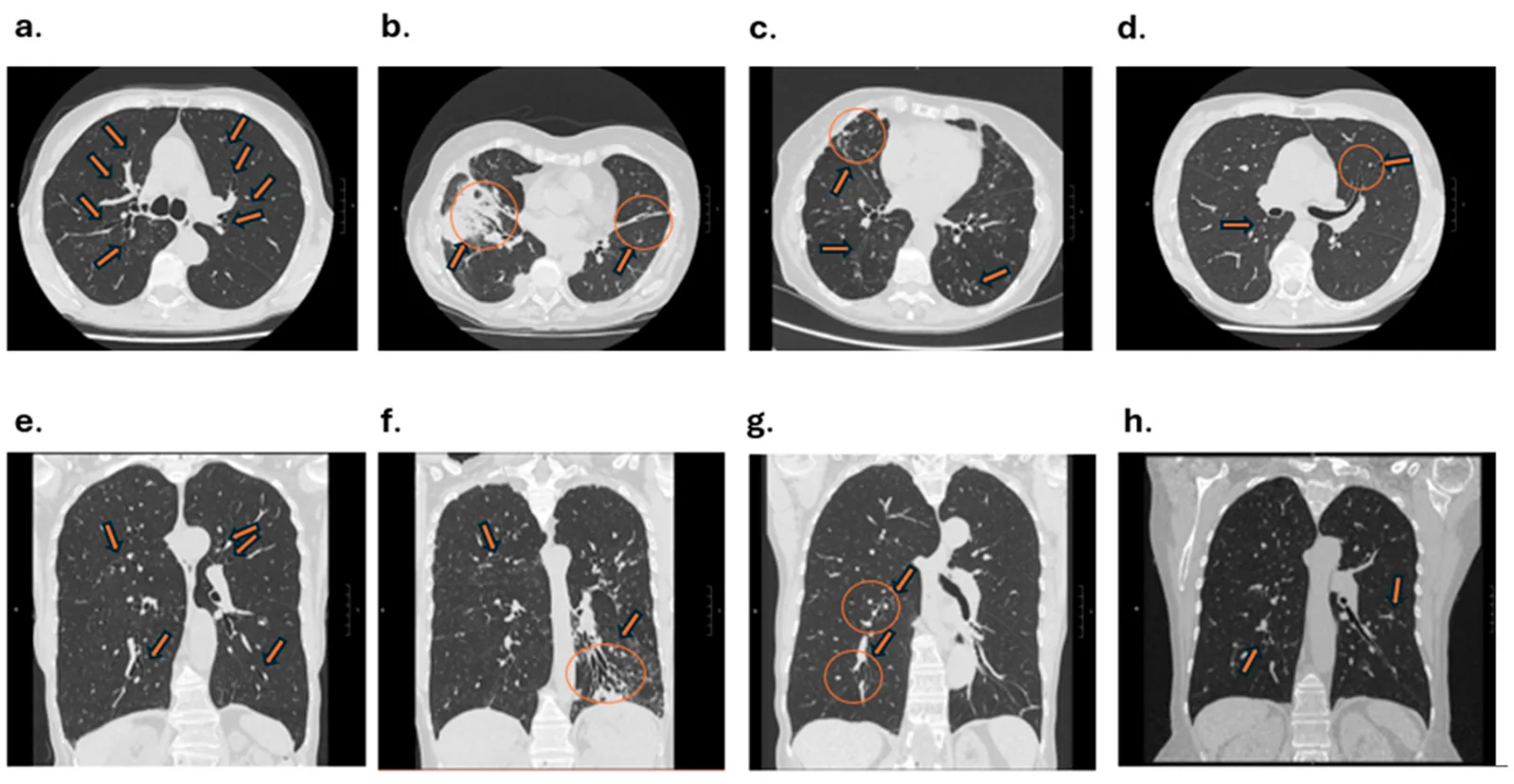
Bronchiectasis in various rheumatologic diseases and in non-rheumatologic patients. From left to right: Small Vessel Vasculitis (panels (**a**,**e**)), Rheumatoid Arthritis (panels (**b**,**f**)), Sjögren’s Disease (panels (**c**,**g**)) and non-rheumatologic patient’s bronchiectasis (panels (**d**,**h**)). Orange arrows and circles indicate bronchiectasis.

The relationship between systemic autoimmune rheumatic diseases and bronchiectasis is clinically relevant, but the available evidence remains difficult to interpret. The literature does not support a single prevalence figure for bronchiectasis across rheumatic diseases. RA has the strongest evidence for bronchiectasis not related to traction in ILD, and Sjögren disease and MPO-AAV/MPA also show consistent features. SSc can include non-traction bronchiectasis, but the frequency and clinical burden remain uncertain; in SLE, current evidence is largely based on diagnostic codes. In inflammatory myopathies, bronchial dilatation is reported chiefly as part of fibrotic ILD. Differences in CT use, symptoms, denominators, and ILD exclusion mean that the available estimates should not be averaged or compared as if they measured the same condition [17,19,20,24,25,26,27,28,29,34,35,39,40,41,42,43] (Figure 4).

A major problem is the mismatch between clinical and radiological findings. Bronchial dilatation on HRCT may be mild, incidental, or entirely explained by fibrosis; conversely, patients with limited dilatation may have substantial sputum, bronchiolitis, infection, or airflow obstruction. Direct review of the images is therefore preferable to reliance on the report alone. The relation of dilated bronchi to fibrosis, together with airway-centred findings, symptoms, exacerbations, and microbiology, usually permits classification as traction bronchiectasis, a separate airway disorder, a mixed pattern, or an indeterminate finding [1].

Where bronchiectasis is clinically active, it should not be treated as an incidental CT observation. Impaired clearance, infection, and neutrophilic inflammation can sustain a self-reinforcing cycle of airway injury, while immunosuppression may increase susceptibility to pulmonary infection and subsequent exacerbations. RA cohorts show increased respiratory morbidity and, depending on the comparator, excess mortality [9,10,11,31,32]. Sjögren-associated bronchiectasis is linked to recurrent infection and slowly progressive airway abnormalities [17,41], and AAV-associated bronchiectasis often persists despite control of vasculitis [19]. These observations also support the view that airway damage may continue independently of parenchymal fibrosis or systemic disease activity.

Reflux and aspiration deserve consideration, but the evidence is weaker than their frequent mention in clinical reviews might suggest [23]. Hiatus hernia was more common in the older Sjögren cohort [17], whereas oesophageal dysmotility was almost universal in the SSc HRCT cohort and could not explain who developed bronchiectasis [20]. Reflux, dysphagia, and aspiration should therefore be investigated when the history or imaging suggests them, but they should not automatically be assigned as the cause. We found no evidence that acid suppression or antireflux surgery alters bronchiectasis outcomes in these diseases.

There is equally little direct evidence to guide antirheumatic treatment. No randomised trial has compared DMARDs, biological agents, or JAK inhibitors specifically in patients with both a rheumatic disease and bronchiectasis. The available treatment studies are retrospective and vulnerable to treatment selection and mixed lung phenotypes [11,12,13]. Antirheumatic therapy should therefore be chosen to control the systemic disease, while bronchiectasis is managed according to respiratory guidelines [7]. Prior exacerbations, chronic airway infection, Pseudomonas, nontuberculous mycobacteria, immunoglobulin concentrations, and cumulative glucocorticoid exposure are relevant to that decision. Across the diseases reviewed, no study directly determined whether improved rheumatic disease control reduces bronchiectasis exacerbations or stabilises airway damage.

B-cell-depleting therapy with rituximab, which is widely used in RA, AAV, and other SARDs, warrants specific consideration. Long-term rituximab treatment can cause secondary hypogammaglobulinaemia through sustained B-cell and plasma cell depletion [45], and bronchiectasis has been reported as an adverse event of this therapy [46]. In patients with both a SARD and bronchiectasis who are receiving or being considered for rituximab, IgG concentrations should be measured before treatment and monitored periodically [45]. In patients with documented IgG deficiency and recurrent respiratory infections, immunoglobulin replacement therapy may be indicated as an adjunct to bronchiectasis-specific management, independent of the antirheumatic regimen [7].

The same caution applies to screening. In a patient with a known rheumatic disease, chronic sputum, recurrent lower respiratory infections, haemoptysis, or otherwise unexplained spirometric abnormalities should prompt review of HRCT images and sputum microbiology rather than automatic attribution to ILD. Conversely, autoimmune testing in bronchiectasis is most useful when guided by extrapulmonary features and interpreted with rheumatological input. In a referral-centre cohort of 468 patients with bronchiectasis, 31.4% had at least one positive autoimmune test, but a connective tissue disease was diagnosed in 5.6%; RA accounted for 46% of those diagnoses [47]. This shows that targeted evaluation can be useful but does not establish a benefit from universal untargeted screening.

It should be noted that the cohort of Stainer et al. [47] was a referral-centre population with a higher pretest probability of SARD than unselected bronchiectasis populations, so the yield of autoimmune testing in less selected settings is likely lower. The finding that 31.4% of patients had at least one positive autoimmune test while only 5.6% received a CTD diagnosis also underscores that positive serology requires clinical integration with rheumatological expertise and should not automatically prompt treatment. Autoimmune screening in bronchiectasis remains most useful when guided by extrapulmonary features such as arthralgia, xerostomia, xerophthalmia, skin changes, or Raynaud phenomenon, and when interpreted in multidisciplinary consultation.

The lack of evidence on whether treatment of SARDs influences bronchiectasis should be considered in light of the emerging concept of disease activity. Bronchiectasis is increasingly regarded as a chronic inflammatory disease, and disease activity refers to the current intensity of potentially modifiable processes, including daily symptoms, airway inflammation, infection, and exacerbation risk [8,48]. This distinction is familiar in rheumatology. Structural bronchial dilatation may be irreversible, but inflammatory activity may still be modifiable. Effective control of the systemic disease could therefore reduce bronchiectasis activity in selected patients, although this has not been demonstrated. Prospective studies and dedicated trials should measure rheumatic and bronchiectasis disease activity in parallel and determine whether specific immunomodulatory treatments improve respiratory outcomes without increasing infection risk. This review has several limitations. It was designed as a structured narrative review rather than a systematic review, and the evidence consists mainly of retrospective cohorts, administrative data, and heterogeneous imaging studies. Central HRCT review, clinical definitions, microbiology, and prospective exacerbation data were often absent. Future cohorts should report traction, non-traction, mixed, and indeterminate bronchial dilatation separately; record symptoms and microbiology longitudinally; and use both rheumatic disease and bronchiectasis comparison groups. Treatment exposure should be analysed over time rather than treated as a fixed baseline characteristic.

## 5. Conclusions

Bronchiectasis in systemic autoimmune rheumatic diseases cannot be inferred from bronchial dilatation on CT alone. A clinically meaningful diagnosis requires respiratory symptoms or recurrent infection together with compatible imaging, and the relationship of the abnormal bronchi to fibrotic lung must be examined. The evidence for a separate airway disorder is strongest in RA, Sjögren disease, and MPO-AAV/MPA; it is less secure in SSc and SLE, while most reported bronchial dilatation in inflammatory myopathies is traction-related. When both airway disease and ILD are present, each requires appropriate assessment and treatment. Prospective studies and dedicated trials should determine whether better control of the coexisting SARD reduces bronchiectasis disease activity, even if structural airway damage persists, and define how infection risk should influence immunomodulatory therapy.

## Figures and Tables

**Table 1:** Evidence for bronchiectasis as a distinct airway manifestation across systemic autoimmune rheumatic diseases.

Disease	Representative Evidence	Separation from Traction Bronchiectasis	Interpretation
Rheumatoid arthritis	HRCT meta-analyses, an ILD-excluded case–control study and outcome cohorts, and national coded cohorts [9,10,24,25,26,27,28,29,30,31,32]	Explicit in selected cohorts, but absent or incomplete in many prevalence and claims studies	Strongest evidence for clinically relevant bronchiectasis, supported by studies in which coexisting ILD was excluded through HRCT review
Sjögren disease	A CT-confirmed airway cohort and an earlier bronchiectasis cohort reporting recurrent infection [17,39,40,41]	Airway disease was specifically characterised, although studies were retrospective and CT was not used for population screening	Supports a combined large- and small-airway manifestation distinct from ILD
ANCA-associated vasculitis	Meta-analysis and MPO-AAV/MPA cohorts; bronchiectasis may precede vasculitis and often persists after treatment [18,19,38,44]	ILD and traction bronchiectasis were not consistently excluded across studies	Supports a separate airway manifestation, particularly in MPO-AAV/MPA, but mixed radiological patterns remain possible
Systemic sclerosis	An HRCT-reviewed cohort separating traction changes and a nationwide coded cohort [20,33,34]	Explicit in the HRCT cohort; unavailable in the claims study, although results persisted after exclusion of ILD codes	Non-traction bronchiectasis can occur independently of ILD, but its population frequency and clinical burden remain uncertain
Systemic lupus erythematosus	A nationwide coded cohort showing increased incidence and adverse outcomes; earlier evidence was sparse [16,35]	No central HRCT review or clinical phenotyping in the nationwide study	An association with coded bronchiectasis is supported, but a disease-specific airway manifestation is not yet established
Inflammatory myopathies	Imaging reports and small cohorts describe bronchial dilatation mainly within fibrotic ILD [42,43]	Bronchial dilatation is predominantly spatially related to fibrosis	Current evidence mainly represents traction bronchiectasis
Mixed connective tissue disease	No disease-specific contemporary cohort adequately characterised non-traction bronchiectasis	Not assessable	Insufficient evidence

Abbreviations: AAV, ANCA-associated vasculitis; ANCA, anti-neutrophil cytoplasmic antibody; CT, computed tomography; ILD, interstitial lung disease; MPA, microscopic polyangiitis; MPO, myeloperoxidase; RA, rheumatoid arthritis; SARD, systemic autoimmune rheumatic disease; SLE, systemic lupus erythematosus; SSc, systemic sclerosis.

## Data Availability

The original contributions presented in this study are included in the article/Supplementary Material. Further inquiries can be directed to the corresponding author.

## Supplementary Materials

The following supporting information can be downloaded at https://www.mdpi.com/article/10.3390/diagnostics16213550/s1, Supplementary Table S1: Study-level evidence for bronchiectasis as a distinct airway manifestation across systemic autoimmune rheumatic diseases.

## Abbreviations

The following abbreviations are used in this manuscript

DMARDs	Disease-modifying antirheumatic drugs
RA	Rheumatoid Arthritis
RF	Rheumatoid Factor
CCP	Cyclic Citrulinated Peptides
AAV	ANCA-associated vasculitis
MPA	Microscopic polyangiitis
ANCA	Anti-Neutrophil Cytoplasm Antibodies
MPO	Myeloperoxidase
PR3	Proteinase 3
SSc	Systemic sclerosis
SARD	Systemic Autoimmune Rheumatoid Disease
MCTD	Mixed Connective Tissue Disease
CT	Computed tomography
HRCT	High Resolution Computed Tomography
